# Does tear size influence factors associated with early retear, satisfaction, and functional outcomes after arthroscopic rotator cuff repair?

**DOI:** 10.1371/journal.pone.0350091

**Published:** 2026-05-22

**Authors:** Ala’ Hawa, Ahmed F. Hawwa, James Bilbrough, Victor Chen, Mina Shenouda, George A. C. Murrell

**Affiliations:** 1 Orthopedics Division, Department of Surgery, Faculty of Medicine, Yarmouk University, Irbid, Jordan; 2 Faculty of medicine, Biology and health, University of Manchester, Manchester, United Kingdom; 3 Orthopedic Research Institute, St George Hospital Campus, University of New South Wales, Kogarah, New South Wales, Australia; Shanghai Jiao Tong University Medical School Affiliated Ruijin Hospital, CHINA

## Abstract

**Background:**

Determinants of structural retear, patient satisfaction, and functional outcomes after arthroscopic rotator cuff repair may vary by tear size and influence surgical decision-making.

**Purpose:**

To determine whether tear size influences factors associated with early structural retear, postoperative satisfaction, and functional outcomes after arthroscopic rotator cuff repair.

**Study design:**

Cohort study; Level of evidence, 3.

**Methods:**

A total of 1,166 primary arthroscopic rotator cuff repairs were categorized by mediolateral tear size as small (≤10 mm), medium (11–29 mm), or large (30–50 mm). At 6 months, patients underwent standardized ultrasound evaluation for structural retear and completed a 5-point satisfaction scale. Shoulder strength and range of motion were measured. Multivariable regression analyses were performed separately for each tear size group.

**Results:**

Associations with structural retear differed by tear size. In small tears, older age, longer operative time, greater anteroposterior tear dimension and preoperative stiffness were associated with increased retear risk, while anchors number showed no association. In medium tears, older age, larger anteroposterior tear dimension, and greater preoperative supraspinatus strength were associated with retear risk, whereas greater number of anchors reduced this risk. In large tears, retear was associated with factors including age, sex, range of motion, and pain at rest, with no association with anchors number. Satisfaction also varied by tear size: greater anchors number was associated with higher satisfaction in small tears, whereas in medium and large tears satisfaction was related to shoulder-related factors. The number of anchors was not consistently associated with postoperative strength or range of motion.

**Conclusion:**

Factors associated with re-tear, satisfaction and functional outcomes vary by tear size. Greater number of anchors reduced the odds of retear only in medium tears and showed minimal association with functional outcomes, supporting tear size–specific counselling and surgical planning.

## Introduction

Rotator cuff tears (RCTs) are considered as one of the most common causes of shoulder pain mainly in individuals older than 60 years of age, they can lead to shoulder disability and impaired upper extremity function [1,2]. Arthroscopic rotator cuff repair (ARCR) is becoming the standard method of management for full-thickness tears, offering reliable pain relief, shorter hospital stays, and improved patient outcomes [3–6]. Despite these advantages, repair failure is still considered a clinical problem, with reported retear rates ranging from 11% to more than 90%, based on patients and tears characteristics, tissue quality and surgical technique used for repair [6–10].

Several studies have identified factors that influence tendon healing and postoperative outcomes following ARCR. Increasing age and larger initial tear size are associated with higher retear rates and less favorable outcomes [5,7,9, 11–13]. In addition, poor tissue quality, fatty infiltration, and chronicity of the tear have been shown to affect tissue healing potential and increase the risk of structural failure [5,14–16]. These findings suggest that different factors would affect tendon healing and show the importance of patient and tear-specific factors in determining postoperative outcomes.

In addition to these factors, surgical variables have drawn increasing attention. In particular, the optimal number of suture anchors required to achieve intact repair is still debatable. Biomechanical studies suggest that increasing the number of anchors or using more complex repair constructs may improve initial fixation strength and footprint coverage [17–20]; however, clinical studies have not demonstrated constant reduction in retear rates with greater number of anchors, particularly in small- and medium-sized tears [21–25]. Previous work from our group suggested that greater number of anchors in small tears may prolong operative time without improving structural integrity or patient reported outcomes [21]. How the association between the number of anchors and repair outcomes differs across tear size groups, however, remains incompletely defined. In addition, the relationship between the number of anchors and other potential modifiers—such as preoperative stiffness, range of motion, muscle strength, and sex— remains incompletely explored within tear size–specific analyses in most previous studies [11,26,27]. Identifying whether and when the number of anchors associated with early outcomes may help inform intraoperative decision-making and preoperative patient counseling.

In addition to structural integrity, postoperative patient satisfaction represents an important outcome following ARCR. Preoperative pain characteristics, shoulder range of motion, and muscle strength have all been shown to affect postoperative functional outcomes and patient-reported outcomes after rotator cuff repair [28,29]. However, whether factors associated with patient satisfaction align with those associated with structural healing, or differ according to tear size, remains unclear. Moreover, there is limited data evaluating patient-reported satisfaction together with structural healing and objective functional outcomes across different tear sizes.

The aim of this study was to identify patient-specific, tear-related and surgical factors associated with early structural retear, postoperative patient satisfaction, and early functional outcomes (strength and range of motion) after arthroscopic rotator cuff repair using a tear size–stratified analysis. By examining these associations separately in small, medium, and large tears, our aim was to clarify tear size–specific risk profiles through structural, patient-reported, and functional outcomes to better inform future surgical decision-making and patient counseling.

## Methods

### Study design

This study was performed retrospectively to identify the factors associated with early structural retear, patient satisfaction and patient reported outcome after arthroscopic rotator cuff repair and to examine tear size–specific associations including number of anchors, patient, tear and surgical-related characteristics using a structured multivariable logistic regression. Patients were classified according to mediolateral rotator cuff tear dimension into small (≤10 mm), medium (11–29 mm), and large (30–50 mm) tear groups. Medioloateral tear dimension was used as the prespecified stratification variable in our institutional database and surgical workflow. Because tear size is multidimensional and anteroposterior tear extent is also clinically relevant, anteroposterior tear dimension was retained as a continuous covariate in all multivariable analyses to evaluate its independent association with outcomes. All analyses were performed separately within each tear size group. **The primary outcome** was early structural retear, defined as evidence of tendon defect or non-healing on ultrasound assessment performed at 6 months postoperatively by an experienced musculoskeletal sonographer. **The secondary outcome** was postoperative patient satisfaction, assessed at 6 months using a 5-level ordinal satisfaction scale. For the multivariable analyses, satisfaction was dichotomized as Good (scores 3–4) versus Poor (scores 0–2) after collection using modified L’Insalata Shoulder Rating Scale [30]. This threshold-based approach for the interpretation of patient satisfaction is consistent with prior work defining satisfaction cut points for clinically meaningful interpretation after rotator cuff repair [31]. In addition, an ordinal logistic regression analysis using the full scale was also performed as a sensitivity analysis.

For each tear size group, tear size–stratified multivariable logistic regression models were built for both outcomes. Covariates included the number of anchors and clinically relevant patient, tear and surgical-related factors: age, sex, operative time, anteroposterior tear dimension, tissue quality (binary), pre-operative stiffness, passive range of motion (abduction and external rotation), preoperative muscle strength (supraspinatus and external rotation), preoperative pain (at rest and during activity), and symptom duration. Sensitivity analysis was performed, pooled models including tear size × anchors number interaction terms were fitted to assess whether the association between the number of anchors and outcome differed statistically by tear size.

Tissue quality was graded intraoperatively using a four-point Likert scale (excellent, good, fair, poor) based on tendon appearance, thickness, and handling properties. For regression analyses, tissue quality was dichotomized into “good/excellent” versus “fair/poor” to improve interpretability and statistical stability. Variable selection was based on prior literature and clinical relevance.

Continuous variables were additionally explored using binned chi-square or Fisher’s exact tests, as appropriate to evaluate potential clinically relevant thresholds. Variables such as age and anteroposterior tear size were divided into quartiles, and adjacent bins were compared to identify inflection points where outcome rates differed. These threshold analyses were exploratory and intended to aid clinical interpretation; identified thresholds were not used to redefine variables or modify the multivariable regression models. Models were designed to identify statistical associations rather than to develop predictive tools.

### Ultrasound assessment of cuff integrity

All postoperative ultrasound examinations were performed at 6 months by an experienced musculoskeletal sonographer (Graduate Diploma in Medical Ultrasound) using a high-resolution linear transducer (9–15 MHz), with the focal zone centered at the tendon footprint. Standardized long- and short axis sweeps were performed with dynamic abduction and internal/external rotation. The examination protocol included systematic assessment of the supraspinatus, infraspinatus, and teres minor tendons. Dynamic imaging was used to differentiate imaging artifacts from true tendon discontinuities. The sonographer was blinded to patients’ clinical details and repair construct.

### Criteria for Retear

A structural retear was defined as a visible focal discontinuity of the repaired tendon fibers or substantial thinning (<50% of expected tendon thickness) at the footprint site, confirmed in two orthogonal imaging planes. When findings were equivocal, additional dynamic assessments were performed. Prior studies using this standardized protocol have demonstrated high inter- and intra-observer reliability (κ > 0.8) [32]. Although magnetic resonance imaging provides excellent soft-tissue contrast, ultrasound was selected as a validated and cost-effective modality for detecting early retears when performed using a consistent protocol by an experienced operator.

### Ethics approval

Ethical approval was obtained from the South Eastern Sydney Local Health Network Human Research Ethics Committee–Southern Sector (2019/ETH14049). This study represents a retrospective analysis of prospectively collected intraoperative and clinical data from 1^st^ of January 2008–31^st^ December 2023. The de-identified dataset was accessed for research purposes on 01/09/2025. Researchers had access to limited identifying information during data collection for study purposes only. All identifying information was removed prior to analysis and publication. Data were stored securely on a password-protected server with restricted access.

### Inclusion criteria

Patients were included if they underwent primary arthroscopic repair of a full-thickness rotator cuff tear using one or more anchors, had a minimum follow-up of 6 months and underwent ultrasound assessment of repair integrity at 6 months postoperatively.

### Exclusion criteria

Patients were excluded they had isolated subscapularis tears, irreparable tears, tears with mediolateral diameter greater than 50 mm (massive tears), partial-thickness tears, revision procedures, tears associated with avulsion fractures, repairs augmented with synthetic (PTFE) patches and if they failed to attend the 6-month follow-up visit. Anchor subgroups with fewer than 10 patients within a tear size group were also excluded to ensure models stability.

### Surgical technique

All procedures were performed arthroscopically. Patients received an interscalene block with sedation and were positioned in the upright beach-chair position. A posterolateral portal was used for arthroscopic visualization and an anterolateral portal for instrumentation. Rotator cuff tendons were repaired using an inverted mattress single-row configuration with a suture passer (Opus SmartStitch; Smith & Nephew) and knotless No. 2 polyester braided suture anchors (Opus Magnum No. 2; Smith & Nephew), using either an undersurface (articular-side only) or combined bursal and articular-side technique [33]. No additional concomitant procedures, such as acromioplasty, were performed as part of the surgical treatment in this cohort.

### Postoperative protocol

Patients were discharged on the day of surgery and immobilized in an abduction pillow sling (UltraSling; DJO) for 6 weeks. During the first postoperative week, grip strengthening, elbow range of motion, and pendular shoulder exercises were initiated. From weeks 1–6, passive shoulder flexion, extension, internal rotation, and external rotation exercises were performed. Active shoulder motion and isometric strengthening commenced after 6 weeks. At 3 months postoperatively, patients were allowed to lift objects weighing ≥5 kg and resume overhead activities. Follow-up visits occurred at 1 week, 6 weeks, and 6 months, with ultrasound evaluation of cuff integrity at 6 months, consistent with prior studies [34].

### Data collection

Data were obtained from a prospectively maintained institutional database and analyzed retrospectively. No patient contact occurred during the analysis phase. Rotator cuff tear dimensions (anteroposterior and mediolateral) were measured intraoperatively using the arthroscopic shaver head diameter as a reference, a method previously shown to provide reliable tear size estimation [35]. Operative time was recorded from skin incision to wound closure.

Preoperative variables included patient demographics (age, sex), symptoms duration, pain characteristics (severity at rest and frequency during activity), shoulder range of motion, preoperative stiffness (modified L’Insalata Shoulder Rating Scale), and muscle strength (supraspinatus and external rotation), measured using a handheld dynamometer (HFG 110; Transducer Techniques). Tissue quality was assessed intraoperatively using a four-point Likert scale. Postoperative patient satisfaction was assessed at 6 months using a patient-reported satisfaction scale (modified L’Insalata Shoulder Rating Scale), and structural integrity was assessed using ultrasound at the same time interval.

### Statistical analysis

Continuous variables are reported as mean ± standard deviation or median with interquartile range, as appropriate. Statistical analyses were performed using SPSS version 27.0 (IBM Corp., Armonk, NY) & Python. Statistical significance was defined as p < 0.05 (two-tailed).

Distributional characteristics of continuous variables were assessed before selecting the appropriate test for descriptive group comparisons. Due to the presence of multiple continuous variables that deviated from a normal distribution, Mann–Whitney U tests were used as a consistent nonparametric approach for continuous variables, whereas chi-square or Fisher exact tests were used for categorical variables. These univariable comparisons were descriptive and were not the primary basis for inference; the main study conclusions were derived from the multivariable regression analyses. Figures were generated using GraphPad Prism version 10 (GraphPad Software, San Diego, CA). Multivariable regression analyses were used to evaluate independent associations while adjusting for prespecified covariates, exploratory bin analyses were used to identify clinically interpretable threshold effects, and sensitivity analyses were performed to assess the robustness of the main findings. Multicollinearity among all prespecified covariates included in the main tear size–stratified multivariable models was assessed before model fitting using variance inflation factors within each tear size group. No evidence of problematic multicollinearity was identified, with all VIF values below 2.5 in the main models.

Within each tear size group, multivariable logistic regression models were built to identify factors independently associated with structural retear. Postoperative patient satisfaction was analyzed using tear size–specific multivariable logistic regression models after dichotomization into Good (scores 3–4) versus Poor (scores 0–2). Anchor subgroups with fewer than 10 patients were excluded to ensure model stability.

Postoperative range-of-motion and strength analyses were prespecified as secondary and exploratory functional outcomes and were intended to provide additional clinical context and were not used for the main conclusions

Tear size–stratified models were fit using the same prespecified variables set within each subgroup, and full model outputs with the sensitivity analysis models are provided in S4–S15 Tables.

## Results

### Study population and exclusions

**Fig 1:**
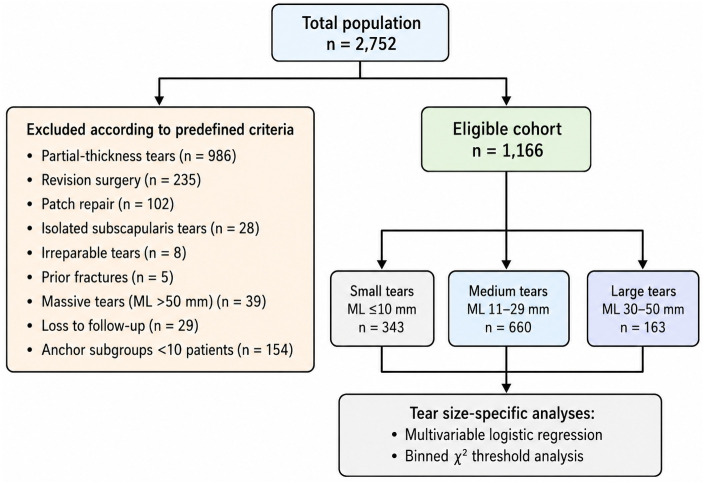
Study cohort flow diagram. Our population started with 2,752 patients who underwent arthroscopic rotator cuff repair, 1,586 were excluded according to the exclusion criteria. The final eligible cohort comprised 1,166 full-thickness repairs, which were stratified by mediolateral tear size into small (≤10 mm), medium (11–29 mm), and large (30–50 mm) tears. Tear size–specific multivariable logistic regression and binned χ² threshold analyses were performed within each subgroup.

2,752 patients underwent arthroscopic rotator cuff repair between January 2008 and December 2023. Of these, 1,586 patients were excluded due to having: partial-thickness tears (n = 986), revision procedures (n = 235), patch-augmented repairs (n = 102), isolated subscapularis tears (n = 28), irreparable tears (n = 8), prior proximal humerus fractures (n = 5), massive tears with a mediolateral (ML) dimension greater than 50 mm (n = 39) or failure to attend the 6-month follow-up visit (n = 29). Anchor subgroups with fewer than 10 patients within a tear size group (n = 154) were excluded *a priori* to reduce instability in multivariable analysis. The final analytic cohort consisted of 1,166 patients with complete clinical, surgical, and imaging data (Fig 1).

### Patient characteristics

Baseline demographics, tear-related, surgical and preoperative clinical characteristics stratified by tear size are summarized in Table 1. The mean age of the patients in the cohort was 61.7 ± 10.6 years, and 58.5% of patients were male. Based on mediolateral tear dimension, tears were classified as small (≤10 mm; n = 343, 29.4%), medium (11–29 mm; n = 660, 56.6%), and large (30–50 mm; n = 163, 14.0%).

**Table 1:** Baseline characteristics by tear size group. Continuous variables are reported as mean ± standard deviation (SD) unless otherwise specified; number of anchors and symptom duration are reported as median (interquartile range [IQR]). Categorical variables are reported as number (percentage).

Characteristic	Small	Medium	Large
**N**	343	660	163
**Age (years)**	60.1 ± 10.4	61.9 ± 10.5	64.1 ± 11.2
**Female sex, n (%)**	163 (47.5%)	266 (40.3%)	55 (33.7%)
**ML tear size (mm)**	9.4 ± 1.5	18.4 ± 3.5	34.9 ± 6.3
**AP tear size (mm)**	14.0 ± 5.8	21.0 ± 7.4	34.5 ± 9.5
**Number of anchors**	2.0 (1.0–2.0)	2.0 (2.0–3.0)	3.0 (3.0–4.0)
**Operative time (min)**	16.3 ± 9.4	19.5 ± 10.6	28.7 ± 14.2
**Good tissue quality, n (%)**	334 (97.4%)	614 (93.0%)	131 (80.4%)
**Preoperative stiffness**	1.8 ± 1.3	1.8 ± 1.3	1.9 ± 1.2
**Passive abduction ROM (deg)**	115.1 ± 39.1	119.9 ± 40.2	105.6 ± 42.3
**Passive external rotation ROM (deg)**	50.0 ± 20.7	51.2 ± 20.9	50.0 ± 24.4
**Supraspinatus strength**	34.5 ± 24.0	35.6 ± 25.4	27.9 ± 20.4
**External rotation strength**	43.8 ± 21.7	45.7 ± 23.7	39.3 ± 23.4
**Pain at rest**	1.9 ± 1.1	1.8 ± 1.0	1.8 ± 1.1
**Pain during activity**	3.5 ± 0.7	3.5 ± 0.7	3.6 ± 0.8
**Symptom duration (days)**	180.5 (114.0–536.0)	180.5 (85.8–537.0)	170.0 (61.5–274.5)

Operative time increased with increasing tear size, and a higher proportion of fair-quality tendon tissue was observed in large tears compared with small and medium tears.

### Structural retear

**Fig 2:**
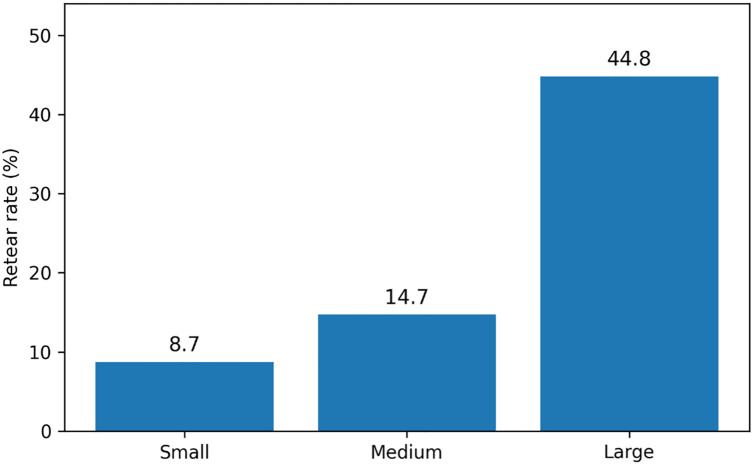
Structural retear rates by tear size. Bar graph demonstrating the proportion of patients with structural retear identified on 6-month postoperative ultrasound, stratified by mediolateral tear size. Retear rates increased with increasing tear size, supporting tear-size–specific evaluation of factors associated with repair failure.

Structural retear rates stratified by tear size are shown in Fig 2. Retear occurred in 8.7% of small tears, 14.7% of medium tears, and 44.8% of large tears.

**Fig 3:**
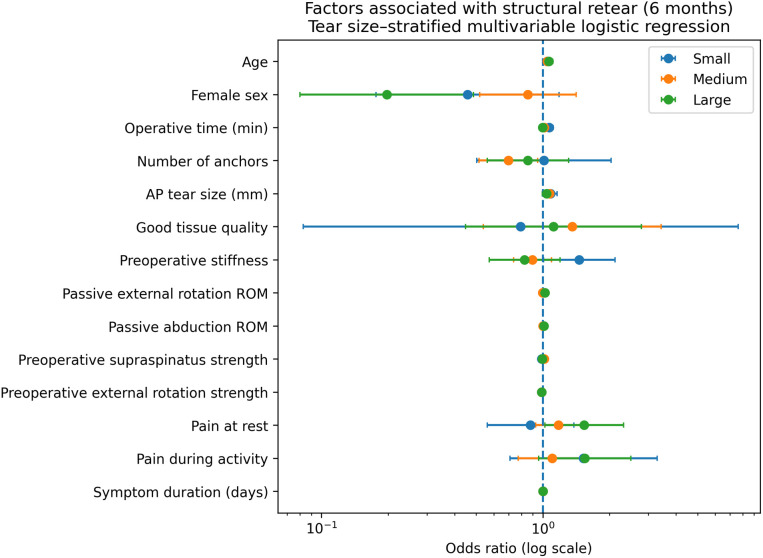
Factors associated with structural retear. Forest plot showing odds ratios (ORs) with 95% confidence intervals from tear size–stratified multivariable logistic regression models evaluating factors associated with structural retear at 6 months following arthroscopic rotator cuff repair. Models were adjusted for demographic, surgical, tear-related, and preoperative clinical variables and fitted separately for small, medium, and large tears. An OR greater than 1 indicates increased odds of retear.

Tear size–stratified multivariable logistic regression analyses evaluating factors associated with structural retear are presented in Table 2 and Fig 3.

**Table 2:** Factors associated with structural retear. Separate models were fitted within each tear size group (Small, Medium, Large). Results are reported as ORs with 95% confidence intervals (CIs) and corresponding p-values. These analyses assess associations and were not intended for prediction.

Factor	Small	Medium	Large
**Age**	**1.05 (1.00–1.09), p = 0.035**	**1.05 (1.02–1.07), p < 0.001**	**1.07 (1.03–1.11), p < 0.001**
**Female sex**	0.46 (0.18–1.18), p = 0.107	0.86 (0.52–1.42), p = 0.545	**0.20 (0.08–0.49), p < 0.001**
**Operative time (min)**	**1.07 (1.03–1.11), p = 0.001**	1.01 (0.99–1.04), p = 0.215	1.00 (0.97–1.03), p = 0.863
**Number of anchors**	1.01 (0.50–2.04), p = 0.973	**0.70 (0.51–0.95), p = 0.021**	0.86 (0.56–1.31), p = 0.47
**AP tear size (mm)**	**1.08 (1.01–1.16), p = 0.017**	**1.07 (1.03–1.11), p < 0.001**	1.04 (0.99–1.09), p = 0.1
**Good tissue quality**	0.79 (0.08–7.63), p = 0.842	1.36 (0.54–3.43), p = 0.515	1.12 (0.45–2.78), p = 0.813
**Preoperative stiffness**	**1.46 (1.01–2.12), p = 0.046**	0.90 (0.74–1.09), p = 0.286	0.83 (0.57–1.19), p = 0.311
**Passive external rotation ROM**	1.02 (0.99–1.04), p = 0.162	1.00 (0.99–1.01), p = 0.872	**1.02 (1.01–1.04), p = 0.01**
**Passive abduction ROM**	1.01 (0.99–1.02), p = 0.24	1.00 (1.00–1.01), p = 0.642	**1.01 (1.00–1.02), p = 0.023**
**Preoperative supraspinatus strength**	0.99 (0.96–1.01), p = 0.301	**1.01 (1.00–1.03), p = 0.026**	0.99 (0.97–1.02), p = 0.679
**Preoperative external rotation strength**	0.99 (0.96–1.02), p = 0.34	0.99 (0.97–1.00), p = 0.141	0.99 (0.96–1.01), p = 0.277
**Pain at rest**	0.88 (0.56–1.38), p = 0.579	1.18 (0.93–1.49), p = 0.178	**1.54 (1.02–2.31), p = 0.04**
**Pain during activity**	1.53 (0.71–3.28), p = 0.279	1.10 (0.78–1.56), p = 0.59	1.55 (0.96–2.49), p = 0.075
**Symptom duration (days)**	1.00 (1.00–1.00), p = 0.714	1.00 (1.00–1.00), p = 0.699	1.00 (1.00–1.00), p = 0.837

In small tears, increasing age, greater AP tear dimension, longer operative time, and greater preoperative stiffness were independently associated with higher odds of retear. In medium tears, older age, larger anteroposterior tear dimension, and greater preoperative supraspinatus strength were independently associated with higher odds of retear, whereas increasing the number of anchors was independently associated with lower odds of retear, representing the only tear size group in which the number of anchors demonstrated a protective association. In large tears, structural retear was independently associated with older age, sex, higher pain at rest, and greater passive range of motion in abduction and external rotation.

In the pooled sensitivity analyses we performed using tear size × anchor number interaction term, formal interaction testing did not show that the association between anchor number and structural re-tear differed significantly across tear-size groups (p = 0.144). However, the subgroup specific pooled estimates remained directionally consistent with the stratified analyses, with a protective association between increasing the number of anchors and retear observed only in medium tears (OR, 0.74; 95% CI, 0.56–0.97; p = 0.028).

### Postoperative patient satisfaction

**Fig 4:**
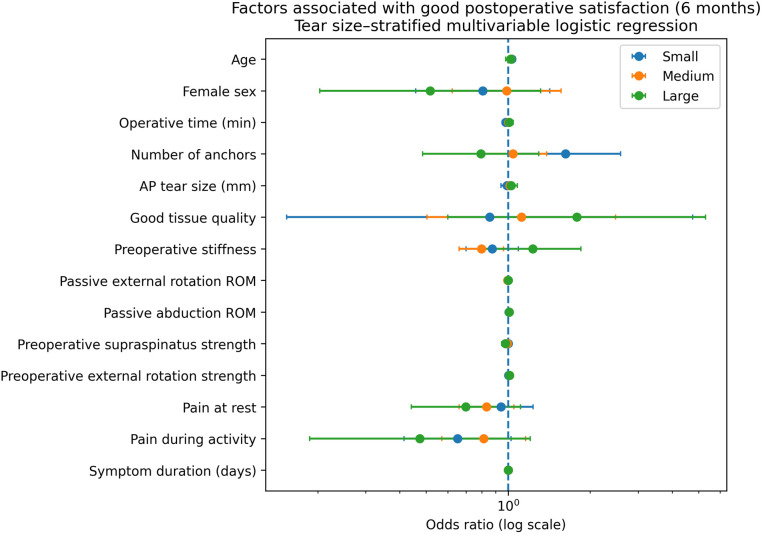
Factors associated with postoperative satisfaction. Forest plot showing odds ratios (ORs) with 95% confidence intervals from tear size–stratified multivariable logistic regression models evaluating factors associated with postoperative satisfaction at 6 months. Satisfaction was dichotomized as Good (scores 3–4) versus Poor (scores 0–2). Models were adjusted for demographic, surgical, tear-related, and preoperative clinical factors. An OR greater than 1 indicates increased odds of good postoperative satisfaction.

Multivariable determinants of postoperative patient satisfaction at 6 months are presented in Table 3 and Fig 4.

**Table 3:** Factors associated with postoperative satisfaction. Separate models were fitted within each tear size group. Results are presented as ORs with 95% CIs and p-values. These models evaluate associations rather than predictive performance.

Factor	Small	Medium	Large
**Age**	**1.03 (1.01–1.06), p = 0.017**	**1.02 (1.00–1.04), p = 0.038**	1.02 (0.98–1.06), p = 0.299
**Female sex**	0.81 (0.46–1.42), p = 0.457	0.99 (0.62–1.56), p = 0.956	0.52 (0.20–1.32), p = 0.167
**Operative time (min)**	0.98 (0.95–1.01), p = 0.169	1.00 (0.98–1.03), p = 0.726	1.01 (0.98–1.04), p = 0.616
**Number of anchors**	**1.62 (1.02–2.59), p = 0.042**	1.04 (0.78–1.38), p = 0.777	0.79 (0.49–1.30), p = 0.355
**AP tear size (mm)**	0.99 (0.94–1.04), p = 0.709	1.01 (0.98–1.05), p = 0.464	1.03 (0.97–1.08), p = 0.33
**Good tissue quality**	0.86 (0.15–4.76), p = 0.859	1.12 (0.50–2.48), p = 0.782	1.79 (0.60–5.31), p = 0.297
**Preoperative stiffness**	0.87 (0.70–1.09), p = 0.231	**0.80 (0.66–0.96), p = 0.018**	1.23 (0.82–1.85), p = 0.313
**Passive external rotation ROM**	1.00 (0.98–1.01), p = 0.735	0.99 (0.98–1.01), p = 0.328	1.00 (0.98–1.02), p = 0.996
**Passive abduction ROM**	1.01 (1.00–1.01), p = 0.075	**1.01 (1.00–1.02), p = 0.003**	1.01 (1.00–1.02), p = 0.16
**Preoperative supraspinatus strength**	1.00 (0.99–1.02), p = 0.911	0.99 (0.98–1.01), p = 0.388	0.98 (0.95–1.01), p = 0.178
**Preoperative external rotation strength**	1.00 (0.99–1.02), p = 0.616	1.01 (1.00–1.03), p = 0.118	1.01 (0.98–1.04), p = 0.414
**Pain at rest**	0.94 (0.72–1.23), p = 0.657	0.83 (0.66–1.05), p = 0.121	0.70 (0.44–1.11), p = 0.129
**Pain during activity**	0.65 (0.42–1.02), p = 0.063	0.81 (0.57–1.16), p = 0.252	0.47 (0.19–1.20), p = 0.117
**Symptom duration (days)**	1.00 (1.00–1.00), p = 0.716	1.00 (1.00–1.00), p = 0.547	1.00 (1.00–1.00), p = 0.545

In small tears, greater number of anchors was independently associated with higher odds of good postoperative satisfaction. In medium tears, greater preoperative stiffness was associated with lower odds of postoperative satisfaction, whereas greater passive abduction range of motion was associated with higher odds of postoperative satisfaction. In large tears, measures of shoulder strength, range of motion and pain demonstrated variable directional associations with postoperative satisfaction, although none reached statistical significance in multivariable analysis.

In the sensitivity analyses performed using ordinal logistic regression with the full 5-level postoperative satisfaction scale, the overall pattern was similar to the primary dichotomized analysis. Greater anchor number was associated with higher postoperative satisfaction in small tears (OR, 1.52; 95% CI, 1.04–2.23; p = 0.031), whereas no clear association was observed in medium or large tears.

### Postoperative functional outcomes (range of motion and strength)

Tear size–stratified multivariable analyses evaluating postoperative functional outcomes, including range of motion and 6-month strength, are summarized in S3 Fig and S3 Table. Number of anchors was not consistently associated with postoperative range-of-motion or strength outcomes across tear size groups. Instead, postoperative function was primarily associated with baseline shoulder characteristics, including preoperative range of motion, preoperative strength, stiffness, sex, and pain characteristics, with the specific associated factors differing by tear size group.

### Risk inflection points

Exploratory threshold analyses identified clinically relevant inflection points for structural retear risk, including an anteroposterior tear dimension greater than 15 mm in small tears and age greater than 69 years in medium tears. These analyses were exploratory in nature and were not used to redefine variables or modify the multivariable regression models.

## Discussion

Previous clinical studies identified several patient, tear and surgical related factors that affect the structural integrity and functional outcome after rotator cuff tears repair [5,6,28]. However, to our knowledge, there are few studies evaluating the association between these factors across different cuff tear sizes. Using tear size–stratified multivariable analyses, we found that the factors associated with early outcomes (structural integrity, satisfaction and functional outcome) including patient characteristics, tear morphology, and preoperative shoulder function showed different associations across different tear groups. Suggesting that early outcomes after rotator cuff repair may vary according to tear size, which may indicate that biological, mechanical, and patient-related factors can contribute differently across tear groups. An important strength of this study is the tear size–stratified analytic approach, which enabled identification of clinically relevant subgroup-specific associations that may have been obscured in a pooled analysis. In particular, greater anchor number was independently associated with lower odds of retear only in medium tears.

**With regard to structural healing.** In small tears; increasing age, larger anteroposterior (AP) tear dimension, longer operative time, and greater preoperative stiffness were independently associated with higher odds of retear. These findings align with previous studies demonstrating that age and tear morphology are important factors associated with structural healing following rotator cuff repair [8,27,36]. In addition, other studies have suggested that simpler fixation strategies may be sufficient for many small tears without increasing the risk of structural failure [21,37]. Together with our findings (no association between the number of anchors and early structural integrity in small tears) this suggests that patients’ characteristics and tear-related factors may play a larger role than fixation construct in the early healing of small tears.

In medium tears, older age, larger AP tear dimension, and greater preoperative supraspinatus strength were associated with higher odds of retear. This association between greater preoperative strength and retear should be interpreted with caution as it may reflect differences in tear chronicity, tissue quality or the presence of compensatory muscle activation rather than a direct association. Interestingly, within this tear size group, increasing the number of anchors was independently associated with lower odds of early retear. This aligns with prior literature suggesting that greater footprint coverage and fixation strength may be beneficial in medium sized tears [17,19,22,25].

Large tears showed a different and less modifiable risk profile. Older age, male sex, greater preoperative pain at rest and greater passive abduction and external rotation range of motion were associated with higher odds of early retear. Multiple studies in the literature reported higher structural failure rates in larger tears and showed the importance of biological and degenerative factors in determining tendon healing potential [3,12,14–16]. The presence of greater pre-operative passive range of motion may indicate capsular laxity or underlying tendon degeneration which may identify patients at increased risk of early repair failure. In addition, because all tears in the present study were repaired using a single-row inverted mattress construct, the high early retear rate observed in larger tears may partly reflect limitations of the repair technique in this subgroup. Accordingly, these findings should be interpreted within the context of single-row repair and may not be directly generalizable to double-row or transosseous-equivalent constructs.

**For factors associated with postoperative satisfaction**. In small tears, greater number of anchors and older age were associated with higher odds of good postoperative satisfaction. Interestingly, the association between the number of anchors and satisfaction occurred despite the absence of concomitant reduction in retear risk, which may suggest that patient-reported satisfaction may reflect early symptom improvement, perceived robustness of repair or patients’ expectations rather than structural healing per se. Previous studies similarly demonstrated that structural integrity and clinical outcomes after rotator cuff repair are not always directly correlated [6,9,10].

In medium tears, greater preoperative stiffness and lower passive abduction range of motion were associated with lower odds of postoperative satisfaction, indicating the influence of baseline shoulder mobility on early patient-reported outcomes in this tear group. In large tears, none of the evaluated factors reached statistical significance in our multivariate analyses, suggesting that satisfaction in larger tears may be influenced by complex interactions between baseline symptoms, patient expectations and perceived functional recovery rather than by isolated surgical variables.

**Regarding functional recovery**. There was no constant association between the number of anchors and early postoperative range of motion or strength throughout tear size groups. Instead, postoperative function was most consistently associated with patients’ baseline characteristics including preoperative range of motion, preoperative strength, stiffness, sex and pain characteristics. Additionally, female sex was consistently associated with lower postoperative strength through several strength measurements. These findings are aligned with previous literature showing that postoperative functional recovery after rotator cuff repair is largely influenced by baseline patient and shoulder characteristics rather than by fixation construct or density alone [5,6,11,24].

These results suggest that increasing the number of anchors was not uniformly associated with better outcomes throughout all tear size groups. In small tears, additional anchors were not associated with better early structural outcomes and adding more anchors may increase the operative time and cost of the surgical intervention. In large tears, the number of anchors was not associated with better structural integrity, satisfaction or functional outcome which may in turn support the concept that biological factors play a major role in determining healing potential in larger tears. In contrast, medium tears were the only tear subgroup in which increasing the number of anchors was associated with better early structural integrity. This tear size–specific pattern may support a more tailored approach to rotator cuff repair in which fixation strategy is chosen based on the biological and biomechanical characteristics of the tear rather than uniformly applying the same anchors principle to all tear sizes [9,21,37,38].

Therefore, our findings show potential opportunities to optimize early postoperative outcomes (structural integrity, satisfaction and functional outcome) through attention to modifiable preoperative factors. Addressing preoperative stiffness, limited range of motion, and pain characteristics before surgery may help improve early postoperative satisfaction, particularly in patients with small and medium tears. Additionally, future studies evaluating biologic augmentation strategies and modified rehabilitation protocols may be particularly important for patients with larger rotator cuff tears, where structural healing appears to be more strongly affected by biological factors.

### Limitations

One limitation of this study is its retrospective design without randomization or blinding, which may introduce the potential for residual confounding despite multivariable adjustment. Follow-up was limited to six months, showing early structural integrity, patient satisfaction and functional outcome but not longer-term outcome. Furthermore, all repairs were performed using a single-row inverted mattress technique, which may limit generalizability to other repair constructs and may have contributed to the high structural failure rate observed in larger tears. The use of musculoskeletal ultrasound rather than magnetic resonance imaging may reduce sensitivity for detecting small or partial defects. However, a standardized imaging protocol and assessment by an experienced musculoskeletal sonographer were used to enhance reliability. Intraoperative tissue quality grading is inherently subjective, although a standardized grading system was applied. In addition, standardized preoperative MRI-based measures of muscle quality, such as fatty infiltration grading, were not available for the full cohort and therefore could not be incorporated into the analyses. Finally, postoperative satisfaction is a patient-reported outcome and may be influenced by baseline expectations and perioperative counseling. Future prospective studies with longer follow-up, standardized imaging protocols, and controlled assessment of patient reported outcomes are needed to confirm the generalizability of our findings.

## Conclusion

Factors associated with early postoperative retear, patient satisfaction and functional outcomes after arthroscopic rotator cuff repair vary according to tear size. Patients’ age, anteroposterior tear dimension, operative time, and selected preoperative clinical characteristics showed tear size–specific associations with early outcomes. The number of anchors was independently associated with lower early retear risk in medium tears but not in small or large tears and showed no consistent association with postoperative functional outcomes. These findings support an individualized, tear size–based approach to surgical planning and patient counseling.

## Supporting information

S1 Fig Distribution of tear size measurements. (A) Distribution of mediolateral (ML) tear size in the analytic cohort, with dashed vertical lines indicating predefined cut points used to define small (≤10 mm), medium (11–29 mm), and large (30–50 mm) tears. (B) Distribution of anteroposterior (AP) tear size stratified by ML tear size group, illustrating variability in AP dimension within each ML category.
(TIFF)

S2 Fig Postoperative satisfaction score distribution by tear size. Bar chart showing the distribution of postoperative satisfaction scores (0–4) at 6 months following surgery, stratified by mediolateral tear size group. Satisfaction scores of 3–4 were categorized as Good and scores of 0–2 as Poor for the primary regression analyses.
(TIFF)

S3 Fig Adjusted association between anchor number and postoperative range of motion. Forest plot showing adjusted β coefficients with 95% confidence intervals for the association between anchor number and postoperative shoulder range-of-motion outcomes (forward flexion, abduction, internal rotation, and external rotation), stratified by tear size. Estimates were derived from tear size–specific multivariable linear regression models and are shown for descriptive purposes.
(TIFF)

S1 Table Expanded baseline characteristics by tear size group. Baseline demographic, tear-related, surgical, and preoperative clinical variables for the analytic cohort. Continuous variables are reported as mean ± SD unless otherwise specified; number of anchors and symptom duration are reported as median (IQR).
(DOCX)

S2 Table Retear and postoperative satisfaction rates by tear size group. Structural retear and postoperative patient satisfaction rates stratified by tear size group. Retear was assessed by 6-month postoperative ultrasound. Postoperative satisfaction was dichotomized as Good (scores 3–4) versus Poor (scores 0–2).
(DOCX)

S3 Table Factors associated with postoperative range of motion and strength. Summary of statistically significant associations from tear size–stratified multivariable linear regression models evaluating postoperative range-of-motion and 6-month strength outcomes. Direction of association is indicated by arrows (↑ positive association, ↓ negative association). These analyses were exploratory and intended to provide functional context.
(DOCX)

S4-15 Tables Full Multivariable Logistic Regression Results. Outcomes were modeled using multivariable logistic regression stratified by tear size group. Odds ratios (OR) are presented per 1-unit increase for continuous predictors; 95% confidence intervals (CI) and p-values are shown.
(DOCX)
