# Supplementary figures and images for: Does tear size influence factors associated with early retear, satisfaction, and functional outcomes after arthroscopic rotator cuff repair?
Source: PLoS One. 2026 May 22;21(5):e0350091. doi: 10.1371/journal.pone.0350091 (PMC13196922; doi:10.1371/journal.pone.0350091)

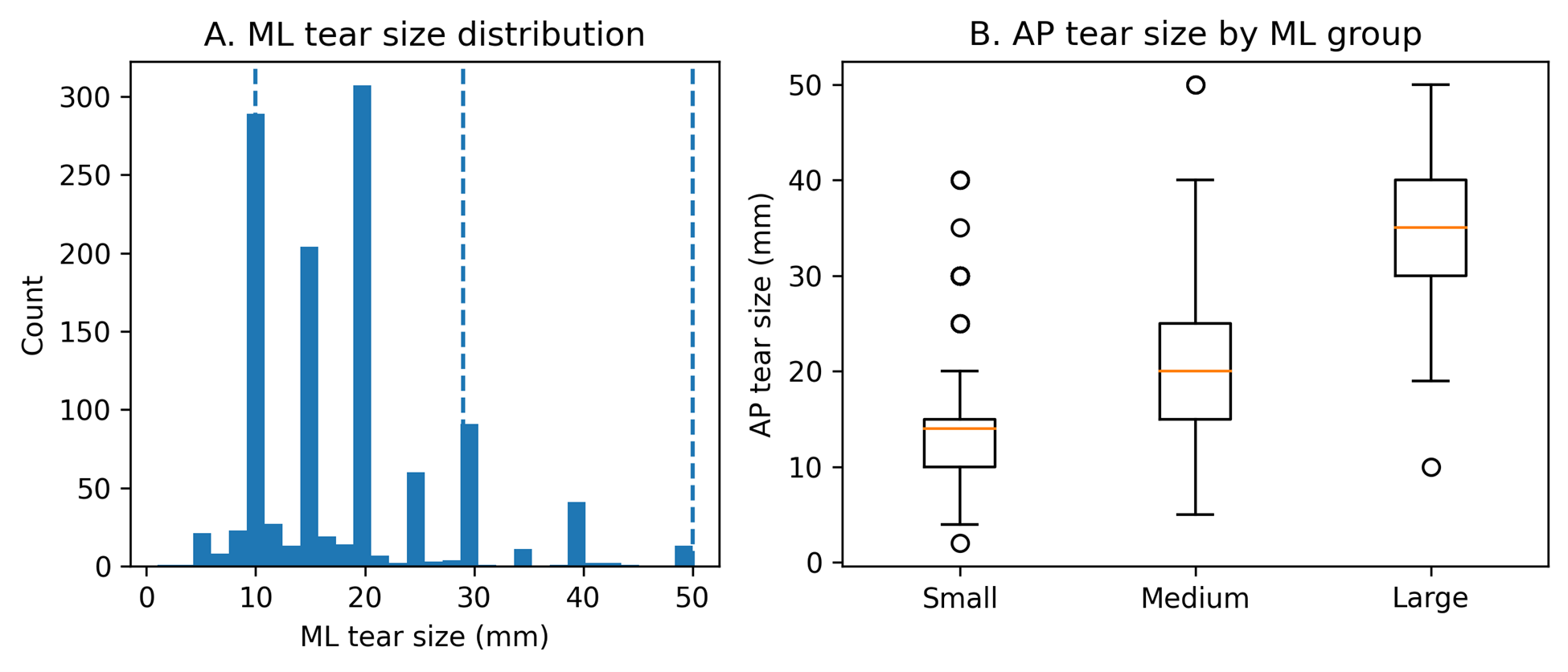

Supplement: S1 Fig — (A) Distribution of mediolateral (ML) tear size in the analytic cohort, with dashed vertical lines indicating predefined cut points used to define small (≤10 mm), medium (11–29 mm), and large (30–50 mm) tears. (B) Distribution of anteroposterior (AP) tear size stratified by ML tear size group, illustrating variability in AP dimension within each ML category. (TIFF) [file pone.0350091.s001.tiff]

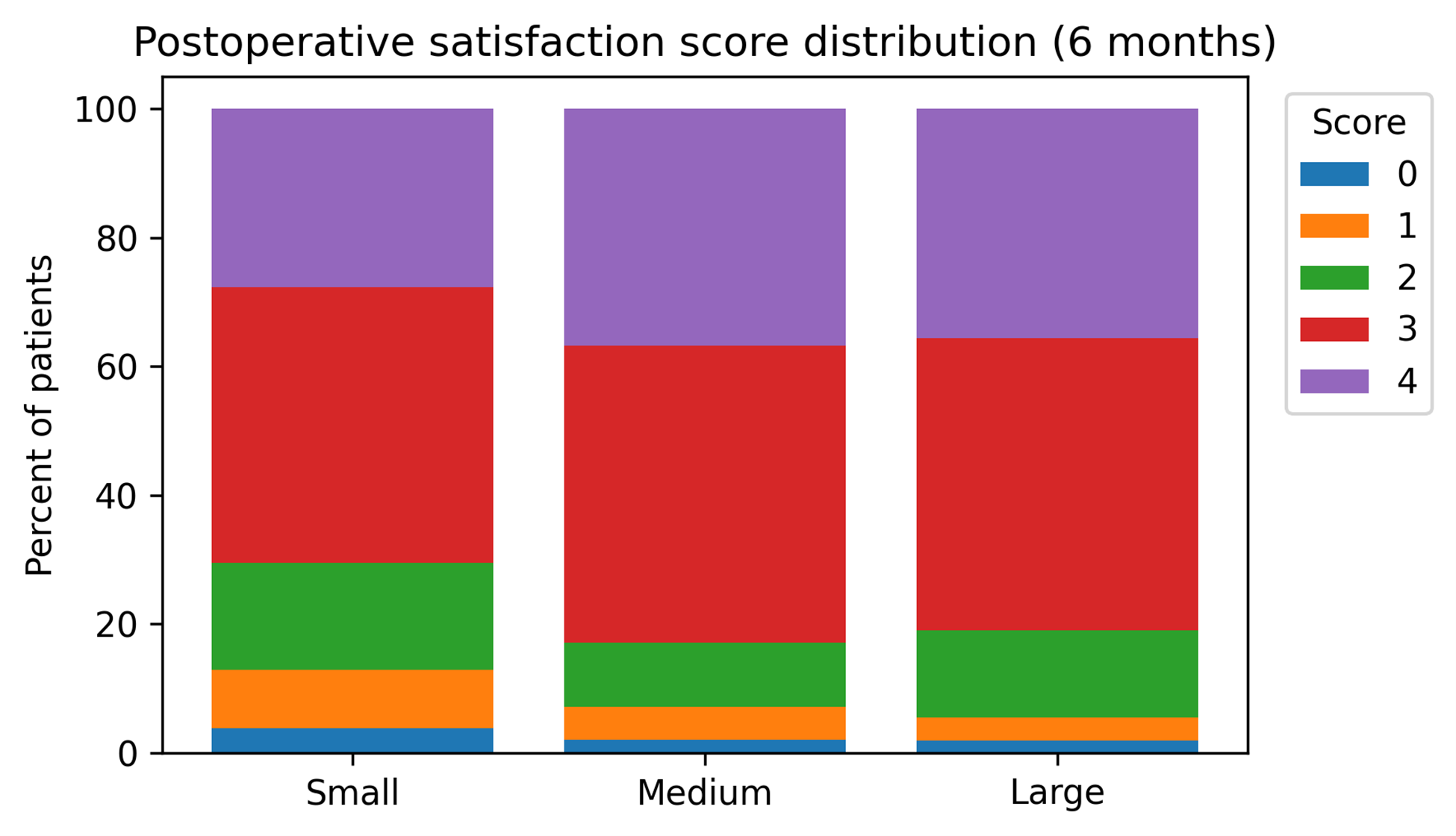

Supplement: S2 Fig — Bar chart showing the distribution of postoperative satisfaction scores (0–4) at 6 months following surgery, stratified by mediolateral tear size group. Satisfaction scores of 3–4 were categorized as Good and scores of 0–2 as Poor for the primary regression analyses. (TIFF) [file pone.0350091.s002.tiff]

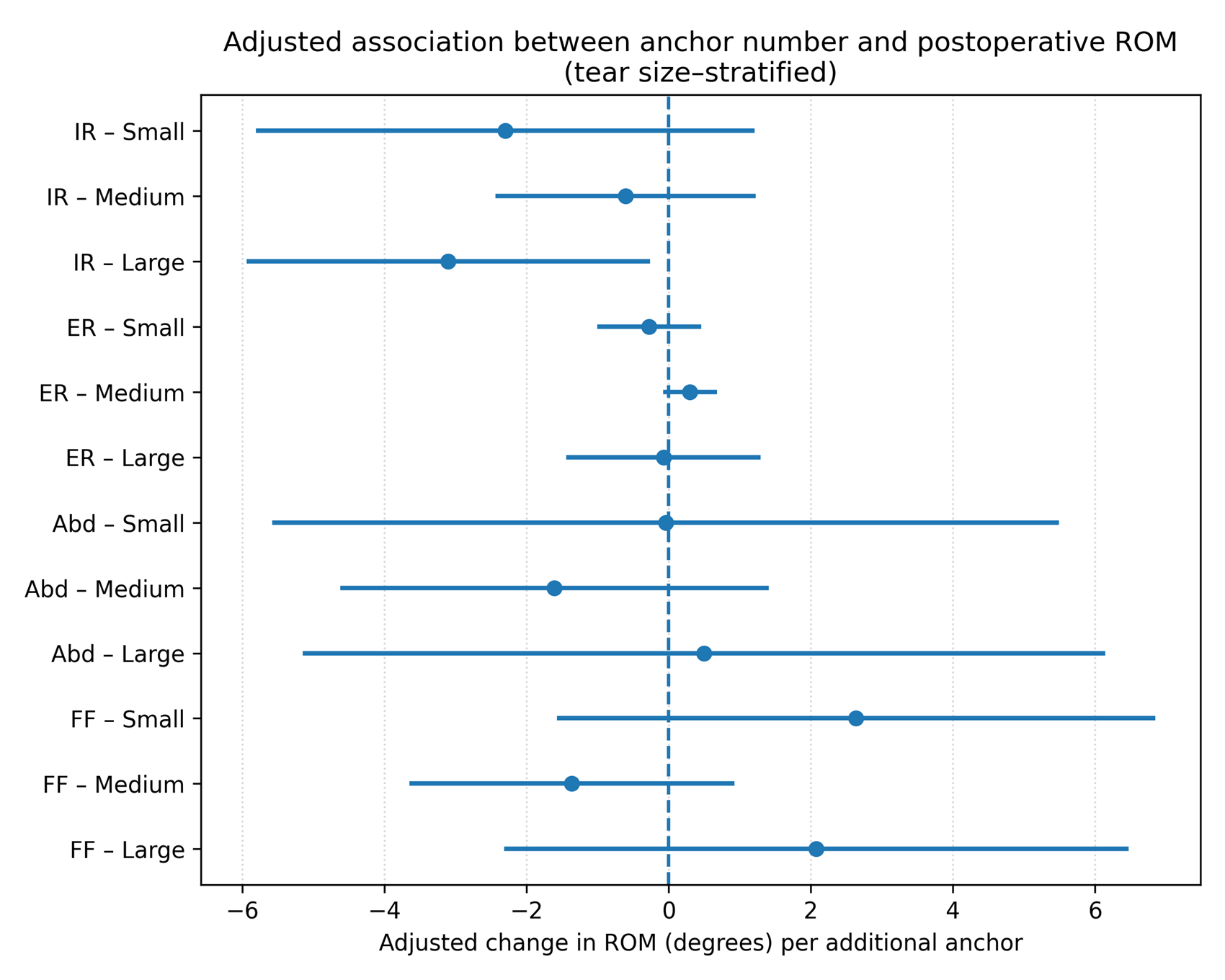

Supplement: S3 Fig — Forest plot showing adjusted β coefficients with 95% confidence intervals for the association between anchor number and postoperative shoulder range-of-motion outcomes (forward flexion, abduction, internal rotation, and external rotation), stratified by tear size. Estimates were derived from tear size–specific multivariable linear regression models and are shown for descriptive purposes. (TIFF) [file pone.0350091.s003.tiff]
