# Supplementary material for: Does tear size influence factors associated with early retear, satisfaction, and functional outcomes after arthroscopic rotator cuff repair?
Source: PLoS One. 2026 May 22;21(5):e0350091. doi: 10.1371/journal.pone.0350091 (PMC13196922; doi:10.1371/journal.pone.0350091)
Supplement: S1 Table — Baseline demographic, tear-related, surgical, and preoperative clinical variables for the analytic cohort. Continuous variables are reported as mean ± SD unless otherwise specified; number of anchors and symptom duration are reported as median (IQR). (DOCX) [file pone.0350091.s004.docx]

| **Characteristic** | **Small** | **Medium** | **Large** |
| --- | --- | --- | --- |
| **N** | 343 | 660 | 163 |
| **Age (years)** | 60.1 ± 10.4 | 61.9 ± 10.5 | 64.1 ± 11.2 |
| **Female sex, n (%)** | 163 (47.5%) | 266 (40.3%) | 55 (33.7%) |
| **ML tear size (mm)** | 9.4 ± 1.5 | 18.4 ± 3.5 | 34.9 ± 6.3 |
| **AP tear size (mm)** | 14.0 ± 5.8 | 21.0 ± 7.4 | 34.5 ± 9.5 |
| **Number of anchors** | 2.0 (1.0–2.0) | 2.0 (2.0–3.0) | 3.0 (3.0–4.0) |
| **Operative time (min)** | 16.3 ± 9.4 | 19.5 ± 10.6 | 28.7 ± 14.2 |
| **Good tissue quality, n (%)** | 334 (97.4%) | 614 (93.0%) | 131 (80.4%) |
| **Preoperative stiffness** | 1.8 ± 1.3 | 1.8 ± 1.3 | 1.9 ± 1.2 |
| **Passive abduction ROM (deg)** | 115.1 ± 39.1 | 119.9 ± 40.2 | 105.6 ± 42.3 |
| **Passive external rotation ROM (deg)** | 50.0 ± 20.7 | 51.2 ± 20.9 | 50.0 ± 24.4 |
| **Supraspinatus strength** | 34.5 ± 24.0 | 35.6 ± 25.4 | 27.9 ± 20.4 |
| **External rotation strength** | 43.8 ± 21.7 | 45.7 ± 23.7 | 39.3 ± 23.4 |
| **Pain at rest** | 1.9 ± 1.1 | 1.8 ± 1.0 | 1.8 ± 1.1 |
| **Pain during activity** | 3.5 ± 0.7 | 3.5 ± 0.7 | 3.6 ± 0.8 |
| **Symptom duration (days)** | 180.5 (114.0–536.0) | 180.5 (85.8–537.0) | 170.0 (61.5–274.5) |

**S1 Table. Expanded baseline characteristics by tear size group.** Baseline demographic, tear-related, surgical, and preoperative clinical variables for the analytic cohort. Continuous variables are reported as mean ± SD unless otherwise specified; number of anchors and symptom duration are reported as median (IQR).
