# Supplementary material for: Does tear size influence factors associated with early retear, satisfaction, and functional outcomes after arthroscopic rotator cuff repair?
Source: PLoS One. 2026 May 22;21(5):e0350091. doi: 10.1371/journal.pone.0350091 (PMC13196922; doi:10.1371/journal.pone.0350091)
Supplement: S2 Table — Structural retear and postoperative patient satisfaction rates stratified by tear size group. Retear was assessed by 6-month postoperative ultrasound. Postoperative satisfaction was dichotomized as Good (scores 3–4) versus Poor (scores 0–2). (DOCX) [file pone.0350091.s005.docx]

| **Tear size group** | **N** | **Retear (%)** | **Satisfaction Good (%)** |
| --- | --- | --- | --- |
| **Large** | 163 | 44.8 | 81.0 |
| **Medium** | 660 | 14.7 | 82.9 |
| **Small** | 343 | 8.7 | 70.6 |

**S2 Table. Retear and postoperative satisfaction rates by tear size group.** Structural retear and postoperative patient satisfaction rates stratified by tear size group. Retear was assessed by 6-month postoperative ultrasound. Postoperative satisfaction was dichotomized as Good (scores 3–4) versus Poor (scores 0–2).
