# Supplementary material for: Does tear size influence factors associated with early retear, satisfaction, and functional outcomes after arthroscopic rotator cuff repair?
Source: PLoS One. 2026 May 22;21(5):e0350091. doi: 10.1371/journal.pone.0350091 (PMC13196922; doi:10.1371/journal.pone.0350091)
Supplement: S3 Table — Summary of statistically significant associations from tear size–stratified multivariable linear regression models evaluating postoperative range-of-motion and 6-month strength outcomes. Direction of association is indicated by arrows (↑ positive association, ↓ negative association). These analyses were exploratory and intended to provide functional context. (DOCX) [file pone.0350091.s006.docx]

| **Outcome** | **Tear size** | **Factor** | **Direction** | **p-value** |
| --- | --- | --- | --- | --- |
| **Post-Op ROM - IR** | Small | Female Sex | ↑ | 0.002 |
| **Post-Op ROM - IR** | Small | Operative Time | ↓ | 0.0 |
| **Post-Op ROM - IR** | Small | Pre-Op Passive ROM - ER | ↑ | 0.0 |
| **Post-Op ROM - IR** | Small | Pre-Op Pain Level - At Rest | ↑ | 0.01 |
| **Post-Op ROM - IR** | Medium | Pre-Op Passive ROM - ER | ↑ | 0.0 |
| **Post-Op ROM - IR** | Large | Number of Anchors | ↓ | 0.032 |
| **Post-Op ROM - IR** | Large | Good Tissue | ↓ | 0.048 |
| **Post-Op ROM - IR** | Large | Pre-Op Pain Level - At Rest | ↓ | 0.039 |
| **Post-Op ROM - ER** | Small | Good Tissue | ↓ | 0.0 |
| **Post-Op ROM - ER** | Small | Pre-Op Passive ROM - ER | ↓ | 0.035 |
| **Post-Op ROM - ER** | Medium | Age | ↑ | 0.01 |
| **Post-Op ROM - ER** | Medium | Operative Time | ↓ | 0.036 |
| **Post-Op ROM - ER** | Medium | Good Tissue | ↓ | 0.0 |
| **Post-Op ROM - ER** | Medium | Pre-Op Passive ROM - ER | ↓ | 0.004 |
| **Post-Op ROM - ER** | Medium | Pre-Op Passive ROM - Abd | ↓ | 0.042 |
| **Post-Op ROM - ER** | Medium | Pre-Op Strength - ER | ↓ | 0.036 |
| **Post-Op ROM - Abd** | Small | Pre-Op Passive ROM - ER | ↑ | 0.021 |
| **Post-Op ROM - Abd** | Small | Pre-Op Passive ROM - Abd | ↑ | 0.0 |
| **Post-Op ROM - Abd** | Medium | Good Tissue | ↑ | 0.0 |
| **Post-Op ROM - Abd** | Medium | Pre-Op Stiffness | ↓ | 0.034 |
| **Post-Op ROM - Abd** | Medium | Pre-Op Passive ROM - Abd | ↑ | 0.0 |
| **Post-Op ROM - Abd** | Medium | Pre-Op Pain Frequency - During Activity | ↓ | 0.017 |
| **Post-Op ROM - Abd** | Large | Age | ↓ | 0.049 |
| **Post-Op ROM - Abd** | Large | Operative Time | ↑ | 0.036 |
| **Post-Op ROM - Abd** | Large | Good Tissue | ↑ | 0.032 |
| **Post-Op ROM - Abd** | Large | Pre-Op Passive ROM - Abd | ↑ | 0.001 |
| **Post-Op ROM - Abd** | Large | Pre-Op Strength - SS | ↑ | 0.029 |
| **Post-Op ROM - Abd** | Large | Pre-Op Strength - ER | ↓ | 0.004 |
| **Post-Op ROM - FF** | Small | Operative Time | ↓ | 0.011 |
| **Post-Op ROM - FF** | Small | Pre-Op Passive ROM - ER | ↑ | 0.001 |
| **Post-Op ROM - FF** | Small | Pre-Op Passive ROM - Abd | ↑ | 0.0 |
| **Post-Op ROM - FF** | Small | Pre-Op Pain Level - At Rest | ↑ | 0.028 |
| **Post-Op ROM - FF** | Medium | Age | ↓ | 0.002 |
| **Post-Op ROM - FF** | Medium | Good Tissue | ↑ | 0.0 |
| **Post-Op ROM - FF** | Medium | Pre-Op Passive ROM - Abd | ↑ | 0.0 |
| **Post-Op ROM - FF** | Medium | Pre-Op Pain Frequency - During Activity | ↓ | 0.007 |
| **Post-Op ROM - FF** | Large | Female Sex | ↑ | 0.042 |
| **Post-Op ROM - FF** | Large | Pre-Op Passive ROM - Abd | ↑ | 0.008 |
| **Post-Op ROM - FF** | Large | Pre-Op Strength - SS | ↑ | 0.017 |
| **Post-Op ROM - FF** | Large | Pre-Op Strength - ER | ↓ | 0.001 |
| **6M Strength - IR** | Small | Female Sex | ↓ | 0.0 |
| **6M Strength - IR** | Small | Operative Time | ↑ | 0.026 |
| **6M Strength - IR** | Medium | Female Sex | ↓ | 0.0 |
| **6M Strength - IR** | Medium | Operative Time | ↑ | 0.02 |
| **6M Strength - IR** | Medium | Pre-Op Strength - ER | ↑ | 0.0 |
| **6M Strength - IR** | Medium | Pre-Op Pain Level - At Rest | ↓ | 0.045 |
| **6M Strength - IR** | Large | Female Sex | ↓ | 0.0 |
| **6M Strength - IR** | Large | Pre-Op Passive ROM - ER | ↓ | 0.019 |
| **6M Strength - IR** | Large | Pre-Op Pain Frequency - During Activity | ↑ | 0.019 |
| **6M Strength - ER** | Small | Female Sex | ↓ | 0.0 |
| **6M Strength - ER** | Small | Operative Time | ↑ | 0.005 |
| **6M Strength - ER** | Small | Pre-Op Strength - ER | ↑ | 0.0 |
| **6M Strength - ER** | Medium | Age | ↓ | 0.039 |
| **6M Strength - ER** | Medium | Female Sex | ↓ | 0.0 |
| **6M Strength - ER** | Medium | Pre-Op Strength - ER | ↑ | 0.0 |
| **6M Strength - ER** | Medium | Pre-Op Pain Level - At Rest | ↓ | 0.03 |
| **6M Strength - ER** | Large | Female Sex | ↓ | 0.001 |
| **6M Strength - ER** | Large | Pre-Op Passive ROM - Abd | ↓ | 0.002 |
| **6M Strength - ER** | Large | Pre-Op Strength - SS | ↑ | 0.049 |
| **6M Strength - ER** | Large | Pre-Op Strength - ER | ↑ | 0.001 |
| **6M Strength - SS** | Small | Female Sex | ↓ | 0.0 |
| **6M Strength - SS** | Medium | Female Sex | ↓ | 0.0 |
| **6M Strength - SS** | Medium | Pre-Op Strength - SS | ↑ | 0.01 |
| **6M Strength - SS** | Large | Female Sex | ↓ | 0.009 |
| **6M Strength - LO** | Small | Female Sex | ↓ | 0.001 |
| **6M Strength - LO** | Small | Anteroposterior Tear Dimension | ↑ | 0.001 |
| **6M Strength - LO** | Small | Pre-Op Strength - ER | ↑ | 0.003 |
| **6M Strength - LO** | Medium | Female Sex | ↓ | 0.0 |
| **6M Strength - LO** | Medium | Operative Time | ↑ | 0.047 |
| **6M Strength - LO** | Medium | Pre-Op Strength - ER | ↑ | 0.029 |
| **6M Strength - LO** | Large | Female Sex | ↓ | 0.0 |
| **6M Strength - ADD** | Small | Female Sex | ↓ | 0.0 |
| **6M Strength - ADD** | Small | Operative Time | ↑ | 0.013 |
| **6M Strength - ADD** | Small | Pre-Op Stiffness | ↓ | 0.023 |
| **6M Strength - ADD** | Medium | Female Sex | ↓ | 0.0 |
| **6M Strength - ADD** | Medium | Pre-Op Strength - ER | ↑ | 0.0 |
| **6M Strength - ADD** | Medium | Pre-Op Pain Level - At Rest | ↓ | 0.05 |
| **6M Strength - ADD** | Large | Female Sex | ↓ | 0.001 |
| **6M Strength - ADD** | Large | Symptom Duration (days) | ↓ | 0.026 |

**S3 Table. Factors associated with postoperative range of motion and strength.** Summary of statistically significant associations from tear size–stratified multivariable linear regression models evaluating postoperative range-of-motion and 6-month strength outcomes. Direction of association is indicated by arrows (↑ positive association, ↓ negative association). These analyses were exploratory and intended to provide functional context.
