# Supplementary material for: Does tear size influence factors associated with early retear, satisfaction, and functional outcomes after arthroscopic rotator cuff repair?
Source: PLoS One. 2026 May 22;21(5):e0350091. doi: 10.1371/journal.pone.0350091 (PMC13196922; doi:10.1371/journal.pone.0350091)
Supplement: S4-15 Tables — Outcomes were modeled using multivariable logistic regression stratified by tear size group. Odds ratios (OR) are presented per 1-unit increase for continuous predictors; 95% confidence intervals (CI) and p-values are shown. (DOCX) [file pone.0350091.s007.docx]

# Supplementary Tables: Full Multivariable Logistic Regression Results

Outcomes were modeled using multivariable logistic regression stratified by tear size group. Odds ratios (OR) are presented per 1-unit increase for continuous predictors; 95% confidence intervals (CI) and p-values are shown.

## Supplementary Table S4. Retear model — Small tears

| **Factor** | **OR (95% CI)** | **p** |
| --- | --- | --- |
| **Age** | 1.05 (1.00–1.09) | 0.035 |
| **Female sex** | 0.46 (0.18–1.18) | 0.107 |
| **Operative time (min)** | 1.07 (1.03–1.11) | 0.001 |
| **Number of anchors** | 1.01 (0.50–2.04) | 0.973 |
| **AP tear size (mm)** | 1.08 (1.01–1.16) | 0.017 |
| **Good tissue quality** | 0.79 (0.08–7.63) | 0.842 |
| **Preoperative stiffness** | 1.46 (1.01–2.12) | 0.046 |
| **Passive external rotation ROM (deg)** | 1.02 (0.99–1.04) | 0.162 |
| **Passive abduction ROM (deg)** | 1.01 (0.99–1.02) | 0.240 |
| **Preoperative supraspinatus strength** | 0.99 (0.96–1.01) | 0.301 |
| **Preoperative external rotation strength** | 0.99 (0.96–1.02) | 0.340 |
| **Pain at rest** | 0.88 (0.56–1.38) | 0.579 |
| **Pain during activity** | 1.53 (0.71–3.28) | 0.279 |
| **Symptom duration (days)** | 1.00 (1.00–1.00) | 0.714 |

## Supplementary Table S5. Retear model — Medium tears

| **Factor** | **OR (95% CI)** | **p** |
| --- | --- | --- |
| **Age** | 1.05 (1.02–1.07) | <0.001 |
| **Female sex** | 0.86 (0.52–1.42) | 0.545 |
| **Operative time (min)** | 1.01 (0.99–1.04) | 0.215 |
| **Number of anchors** | 0.70 (0.51–0.95) | 0.021 |
| **AP tear size (mm)** | 1.07 (1.03–1.11) | <0.001 |
| **Good tissue quality** | 1.36 (0.54–3.43) | 0.515 |
| **Preoperative stiffness** | 0.90 (0.74–1.09) | 0.286 |
| **Passive external rotation ROM (deg)** | 1.00 (0.99–1.01) | 0.872 |
| **Passive abduction ROM (deg)** | 1.00 (1.00–1.01) | 0.642 |
| **Preoperative supraspinatus strength** | 1.01 (1.00–1.03) | 0.026 |
| **Preoperative external rotation strength** | 0.99 (0.97–1.00) | 0.141 |
| **Pain at rest** | 1.18 (0.93–1.49) | 0.178 |
| **Pain during activity** | 1.10 (0.78–1.56) | 0.590 |
| **Symptom duration (days)** | 1.00 (1.00–1.00) | 0.699 |

## Supplementary Table S6. Retear model — Large tears

| **Factor** | **OR (95% CI)** | **p** |
| --- | --- | --- |
| **Age** | 1.07 (1.03–1.11) | <0.001 |
| **Female sex** | 0.20 (0.08–0.49) | <0.001 |
| **Operative time (min)** | 1.00 (0.97–1.03) | 0.863 |
| **Number of anchors** | 0.86 (0.56–1.31) | 0.470 |
| **AP tear size (mm)** | 1.04 (0.99–1.09) | 0.100 |
| **Good tissue quality** | 1.12 (0.45–2.78) | 0.813 |
| **Preoperative stiffness** | 0.83 (0.57–1.19) | 0.311 |
| **Passive external rotation ROM (deg)** | 1.02 (1.01–1.04) | 0.010 |
| **Passive abduction ROM (deg)** | 1.01 (1.00–1.02) | 0.023 |
| **Preoperative supraspinatus strength** | 0.99 (0.97–1.02) | 0.679 |
| **Preoperative external rotation strength** | 0.99 (0.96–1.01) | 0.277 |
| **Pain at rest** | 1.54 (1.02–2.31) | 0.040 |
| **Pain during activity** | 1.55 (0.96–2.49) | 0.075 |
| **Symptom duration (days)** | 1.00 (1.00–1.00) | 0.837 |

## Supplementary Table S7. Satisfaction model — Small tears

| **Factor** | **OR (95% CI)** | **p** |
| --- | --- | --- |
| **Age** | 1.03 (1.01–1.06) | 0.017 |
| **Female sex** | 0.81 (0.46–1.42) | 0.457 |
| **Operative time (min)** | 0.98 (0.95–1.01) | 0.169 |
| **Number of anchors** | 1.62 (1.02–2.59) | 0.042 |
| **AP tear size (mm)** | 0.99 (0.94–1.04) | 0.709 |
| **Good tissue quality** | 0.86 (0.15–4.76) | 0.859 |
| **Preoperative stiffness** | 0.87 (0.70–1.09) | 0.231 |
| **Passive external rotation ROM (deg)** | 1.00 (0.98–1.01) | 0.735 |
| **Passive abduction ROM (deg)** | 1.01 (1.00–1.01) | 0.075 |
| **Preoperative supraspinatus strength** | 1.00 (0.99–1.02) | 0.911 |
| **Preoperative external rotation strength** | 1.00 (0.99–1.02) | 0.616 |
| **Pain at rest** | 0.94 (0.72–1.23) | 0.657 |
| **Pain during activity** | 0.65 (0.42–1.02) | 0.063 |
| **Symptom duration (days)** | 1.00 (1.00–1.00) | 0.716 |

## Supplementary Table S8. Satisfaction model — Medium tears

| **Factor** | **OR (95% CI)** | **p** |
| --- | --- | --- |
| **Age** | 1.02 (1.00–1.04) | 0.038 |
| **Female sex** | 0.99 (0.62–1.56) | 0.956 |
| **Operative time (min)** | 1.00 (0.98–1.03) | 0.726 |
| **Number of anchors** | 1.04 (0.78–1.38) | 0.777 |
| **AP tear size (mm)** | 1.01 (0.98–1.05) | 0.464 |
| **Good tissue quality** | 1.12 (0.50–2.48) | 0.782 |
| **Preoperative stiffness** | 0.80 (0.66–0.96) | 0.018 |
| **Passive external rotation ROM (deg)** | 0.99 (0.98–1.01) | 0.328 |
| **Passive abduction ROM (deg)** | 1.01 (1.00–1.02) | 0.003 |
| **Preoperative supraspinatus strength** | 0.99 (0.98–1.01) | 0.388 |
| **Preoperative external rotation strength** | 1.01 (1.00–1.03) | 0.118 |
| **Pain at rest** | 0.83 (0.66–1.05) | 0.121 |
| **Pain during activity** | 0.81 (0.57–1.16) | 0.252 |
| **Symptom duration (days)** | 1.00 (1.00–1.00) | 0.547 |

## Supplementary Table S9. Satisfaction model — Large tears

| **Factor** | **OR (95% CI)** | **p** |
| --- | --- | --- |
| **Age** | 1.02 (0.98–1.06) | 0.299 |
| **Female sex** | 0.52 (0.20–1.32) | 0.167 |
| **Operative time (min)** | 1.01 (0.98–1.04) | 0.616 |
| **Number of anchors** | 0.79 (0.49–1.30) | 0.355 |
| **AP tear size (mm)** | 1.03 (0.97–1.08) | 0.330 |
| **Good tissue quality** | 1.79 (0.60–5.31) | 0.297 |
| **Preoperative stiffness** | 1.23 (0.82–1.85) | 0.313 |
| **Passive external rotation ROM (deg)** | 1.00 (0.98–1.02) | 0.996 |
| **Passive abduction ROM (deg)** | 1.01 (1.00–1.02) | 0.160 |
| **Preoperative supraspinatus strength** | 0.98 (0.95–1.01) | 0.178 |
| **Preoperative external rotation strength** | 1.01 (0.98–1.04) | 0.414 |
| **Pain at rest** | 0.70 (0.44–1.11) | 0.129 |
| **Pain during activity** | 0.47 (0.19–1.20) | 0.117 |
| **Symptom duration (days)** | 1.00 (1.00–1.00) | 0.545 |

# Postoperative 6-month range of motion and strength — full multivariable linear regression results

Outcomes were modeled using multivariable linear regression stratified by tear size group. Values are β coefficients (95% CI) with p-values. β represents change in outcome per 1-unit increase in the predictor.

## Supplementary Table S10. 6-month postoperative ROM and strength — Small tears

| **Factor** | **6M Passive abduction ROM (deg) β (95% CI)** | **p** | **6M Passive external rotation ROM (deg) β (95% CI)** | **p** | **6M Supraspinatus strength β (95% CI)** | **p** | **6M External rotation strength β (95% CI)** | **p** |
| --- | --- | --- | --- | --- | --- | --- | --- | --- |
| **Age** | 0.11 (-0.31–0.53) | 0.616 | 0.00 (-0.22–0.22) | 0.987 | 0.03 (-0.23–0.29) | 0.806 | -0.13 (-0.36–0.09) | 0.245 |
| **Female sex** | -6.24 (-15.93–3.45) | 0.206 | 6.71 (1.57–11.84) | 0.011 | -13.18 (-19.20–-7.15) | <0.001 | -14.50 (-19.75–-9.25) | <0.001 |
| **Operative time (min)** | -0.03 (-0.51–0.46) | 0.907 | -0.23 (-0.49–0.02) | 0.075 | 0.18 (-0.12–0.49) | 0.231 | 0.38 (0.11–0.64) | 0.005 |
| **Number of anchors** | 4.65 (-3.11–12.40) | 0.239 | 2.24 (-1.87–6.35) | 0.284 | -3.13 (-7.95–1.70) | 0.203 | -0.33 (-4.53–3.87) | 0.877 |
| **AP tear size (mm)** | 0.41 (-0.42–1.24) | 0.333 | 0.05 (-0.39–0.49) | 0.834 | 0.40 (-0.12–0.92) | 0.129 | 0.40 (-0.05–0.85) | 0.085 |
| **Good tissue quality** | 11.64 (-14.89–38.18) | 0.389 | -6.00 (-20.06–8.06) | 0.402 | 4.03 (-12.48–20.54) | 0.631 | -1.59 (-15.98–12.79) | 0.828 |
| **Preoperative stiffness** | -4.88 (-8.68–-1.07) | 0.012 | -3.21 (-5.22–-1.19) | 0.002 | -1.10 (-3.46–1.27) | 0.364 | -1.63 (-3.69–0.44) | 0.122 |
| **Passive external rotation ROM (deg)** | -0.04 (-0.26–0.19) | 0.734 | 0.16 (0.04–0.28) | 0.008 | -0.07 (-0.21–0.07) | 0.332 | -0.05 (-0.17–0.08) | 0.463 |
| **Passive abduction ROM (deg)** | 0.18 (0.05–0.31) | 0.006 | 0.05 (-0.02–0.11) | 0.165 | 0.01 (-0.07–0.09) | 0.811 | -0.01 (-0.08–0.06) | 0.841 |
| **Preoperative supraspinatus strength** | -0.03 (-0.29–0.22) | 0.801 | 0.01 (-0.12–0.15) | 0.838 | 0.12 (-0.04–0.28) | 0.130 | -0.04 (-0.18–0.10) | 0.594 |
| **Preoperative external rotation strength** | 0.06 (-0.22–0.35) | 0.659 | 0.02 (-0.13–0.17) | 0.795 | 0.12 (-0.06–0.30) | 0.185 | 0.34 (0.19–0.50) | <0.001 |
| **Pain at rest** | 1.94 (-2.74–6.62) | 0.415 | 2.30 (-0.18–4.78) | 0.069 | -0.43 (-3.34–2.48) | 0.772 | 0.18 (-2.35–2.72) | 0.886 |
| **Pain during activity** | -10.70 (-17.06–-4.33) | 0.001 | -1.58 (-4.95–1.79) | 0.356 | -1.15 (-5.11–2.81) | 0.568 | -1.93 (-5.38–1.52) | 0.271 |
| **Symptom duration (days)** | 0.00 (-0.00–0.01) | 0.187 | 0.00 (-0.00–0.00) | 0.995 | 0.00 (-0.00–0.00) | 0.388 | 0.00 (-0.00–0.00) | 0.660 |

## Supplementary Table S11. 6-month postoperative ROM and strength — Medium tears

| **Factor** | **6M Passive abduction ROM (deg) β (95% CI)** | **p** | **6M Passive external rotation ROM (deg) β (95% CI)** | **p** | **6M Supraspinatus strength β (95% CI)** | **p** | **6M External rotation strength β (95% CI)** | **p** |
| --- | --- | --- | --- | --- | --- | --- | --- | --- |
| **Age** | -0.26 (-0.53–0.00) | 0.054 | -0.07 (-0.22–0.08) | 0.367 | -0.11 (-0.30–0.07) | 0.233 | -0.17 (-0.33–-0.01) | 0.039 |
| **Female sex** | -1.80 (-7.82–4.21) | 0.556 | 1.97 (-1.45–5.39) | 0.259 | -12.46 (-16.62–-8.29) | <0.001 | -11.80 (-15.41–-8.20) | <0.001 |
| **Operative time (min)** | 0.14 (-0.13–0.42) | 0.300 | -0.07 (-0.23–0.08) | 0.360 | 0.04 (-0.15–0.23) | 0.679 | 0.11 (-0.05–0.28) | 0.170 |
| **Number of anchors** | -0.45 (-4.10–3.21) | 0.811 | -0.41 (-2.48–1.67) | 0.702 | 0.02 (-2.51–2.55) | 0.988 | 0.81 (-1.38–3.00) | 0.467 |
| **AP tear size (mm)** | -0.09 (-0.56–0.38) | 0.709 | 0.07 (-0.20–0.33) | 0.620 | -0.01 (-0.33–0.32) | 0.966 | 0.04 (-0.24–0.32) | 0.779 |
| **Good tissue quality** | -5.97 (-16.71–4.76) | 0.275 | -2.93 (-9.03–3.17) | 0.345 | 6.71 (-0.72–14.14) | 0.076 | 4.93 (-1.50–11.36) | 0.133 |
| **Preoperative stiffness** | -1.55 (-3.94–0.85) | 0.206 | -0.46 (-1.82–0.90) | 0.509 | -1.13 (-2.79–0.53) | 0.181 | 0.41 (-1.02–1.85) | 0.570 |
| **Passive external rotation ROM (deg)** | 0.35 (0.20–0.49) | <0.001 | 0.23 (0.15–0.31) | <0.001 | 0.04 (-0.06–0.13) | 0.466 | 0.07 (-0.01–0.16) | 0.092 |
| **Passive abduction ROM (deg)** | 0.13 (0.05–0.21) | 0.001 | 0.03 (-0.01–0.07) | 0.194 | 0.01 (-0.05–0.06) | 0.842 | -0.02 (-0.07–0.02) | 0.330 |
| **Preoperative supraspinatus strength** | 0.28 (0.12–0.45) | <0.001 | -0.19 (-0.28–-0.09) | <0.001 | 0.15 (0.04–0.26) | 0.010 | 0.00 (-0.10–0.10) | 0.995 |
| **Preoperative external rotation strength** | -0.28 (-0.46–-0.10) | 0.002 | 0.15 (0.05–0.25) | 0.003 | 0.10 (-0.02–0.22) | 0.109 | 0.24 (0.13–0.34) | <0.001 |
| **Pain at rest** | 0.45 (-2.49–3.39) | 0.764 | -0.16 (-1.83–1.51) | 0.848 | -1.62 (-3.66–0.41) | 0.118 | -1.95 (-3.71–-0.19) | 0.030 |
| **Pain during activity** | -5.72 (-9.77–-1.68) | 0.006 | -2.40 (-4.70–-0.10) | 0.041 | -0.66 (-3.46–2.14) | 0.646 | -0.05 (-2.47–2.38) | 0.970 |
| **Symptom duration (days)** | 0.00 (-0.00–0.00) | 0.695 | -0.00 (-0.00–0.00) | 0.346 | 0.00 (-0.00–0.00) | 0.617 | 0.00 (-0.00–0.00) | 0.462 |

## Supplementary Table S12. 6-month postoperative ROM and strength — Large tears

| **Factor** | **6M Passive abduction ROM (deg) β (95% CI)** | **p** | **6M Passive external rotation ROM (deg) β (95% CI)** | **p** | **6M Supraspinatus strength β (95% CI)** | **p** | **6M External rotation strength β (95% CI)** | **p** |
| --- | --- | --- | --- | --- | --- | --- | --- | --- |
| **Age** | -0.14 (-0.69–0.42) | 0.631 | -0.25 (-0.55–0.05) | 0.098 | -0.07 (-0.45–0.31) | 0.714 | -0.09 (-0.40–0.22) | 0.562 |
| **Female sex** | -8.90 (-21.91–4.11) | 0.178 | -2.70 (-9.70–4.31) | 0.448 | -11.95 (-20.85–-3.05) | 0.009 | -12.49 (-19.71–-5.28) | <0.001 |
| **Operative time (min)** | -0.03 (-0.45–0.39) | 0.883 | 0.10 (-0.13–0.32) | 0.404 | 0.05 (-0.24–0.34) | 0.748 | 0.15 (-0.08–0.39) | 0.196 |
| **Number of anchors** | 2.56 (-3.91–9.03) | 0.436 | 1.10 (-2.39–4.58) | 0.535 | -2.26 (-6.69–2.17) | 0.315 | -0.48 (-4.07–3.11) | 0.791 |
| **AP tear size (mm)** | -0.56 (-1.25–0.13) | 0.112 | -0.05 (-0.42–0.33) | 0.807 | -0.26 (-0.74–0.21) | 0.274 | 0.02 (-0.36–0.41) | 0.915 |
| **Good tissue quality** | 3.44 (-11.32–18.19) | 0.646 | -0.13 (-8.08–7.83) | 0.975 | -0.22 (-10.32–9.88) | 0.965 | 2.16 (-6.03–10.34) | 0.603 |
| **Preoperative stiffness** | -4.49 (-10.06–1.09) | 0.114 | -3.09 (-6.10–-0.09) | 0.044 | -2.50 (-6.32–1.31) | 0.197 | -2.03 (-5.12–1.06) | 0.197 |
| **Passive external rotation ROM (deg)** | 0.14 (-0.11–0.39) | 0.281 | 0.06 (-0.08–0.19) | 0.415 | -0.09 (-0.26–0.08) | 0.304 | -0.10 (-0.24–0.04) | 0.143 |
| **Passive abduction ROM (deg)** | 0.02 (-0.14–0.18) | 0.787 | -0.02 (-0.11–0.06) | 0.565 | -0.05 (-0.16–0.05) | 0.334 | -0.14 (-0.23–-0.05) | 0.002 |
| **Preoperative supraspinatus strength** | 0.28 (-0.12–0.69) | 0.171 | -0.02 (-0.24–0.20) | 0.847 | 0.21 (-0.06–0.49) | 0.129 | 0.23 (0.00–0.45) | 0.049 |
| **Preoperative external rotation strength** | -0.22 (-0.57–0.13) | 0.220 | 0.02 (-0.17–0.21) | 0.803 | 0.17 (-0.07–0.41) | 0.156 | 0.34 (0.15–0.54) | <0.001 |
| **Pain at rest** | -0.25 (-6.38–5.89) | 0.936 | -4.30 (-7.60–-0.99) | 0.011 | -0.56 (-4.76–3.64) | 0.791 | -0.38 (-3.78–3.02) | 0.826 |
| **Pain during activity** | 2.80 (-4.37–9.98) | 0.441 | 0.79 (-3.08–4.66) | 0.687 | 0.52 (-4.39–5.44) | 0.833 | 1.99 (-1.99–5.97) | 0.324 |
| **Symptom duration (days)** | -0.00 (-0.01–0.00) | 0.430 | 0.00 (0.00–0.01) | 0.026 | -0.00 (-0.00–0.00) | 0.780 | 0.00 (-0.00–0.00) | 0.399 |

These supplementary tables report the pooled interaction analyses for number of anchors across tear-size groups and the ordinal logistic sensitivity analysis for postoperative satisfaction using the full 5-level scale.

**Supplementary Table S13.** Pooled interaction analyses testing whether the association between number of anchors and outcomes differs by tear size

**S13A. Global interaction tests**

| **Outcome model** | **Interaction tested** | **LR χ²** | **df** | **p value** |
| --- | --- | --- | --- | --- |
| Retear, binary logistic | Tear size × number of anchors | 3.879 | 2 | 0.144 |
| Satisfaction, binary logistic | Tear size × number of anchors | 2.284 | 2 | 0.319 |
| Satisfaction, ordinal logistic (0–4 scale) | Tear size × number of anchors | 3.516 | 2 | 0.172 |

**S13B. Pooled subgroup-specific anchor effects derived from the interaction model**

| **Outcome** | **Tear size** | **OR (95% CI) per additional anchor** | **p value** |
| --- | --- | --- | --- |
| Retear, binary logistic | Small | 1.36 (0.76–2.44) | 0.301 |
| Retear, binary logistic | Medium | 0.74 (0.56–0.97) | 0.028 |
| Retear, binary logistic | Large | 0.85 (0.59–1.21) | 0.366 |
| Satisfaction, binary logistic | Small | 1.38 (0.92–2.07) | 0.123 |
| Satisfaction, binary logistic | Medium | 1.07 (0.83–1.38) | 0.587 |
| Satisfaction, binary logistic | Large | 0.89 (0.58–1.38) | 0.611 |
| Satisfaction, ordinal logistic | Small | 1.43 (1.03–1.99) | 0.032 |
| Satisfaction, ordinal logistic | Medium | 1.03 (0.86–1.22) | 0.756 |
| Satisfaction, ordinal logistic | Large | 1.06 (0.77–1.45) | 0.727 |

**Footnote.** Pooled models adjusted for age, sex, operative time, anteroposterior tear dimension, tissue quality, preoperative stiffness, passive external-rotation ROM, passive abduction ROM, supraspinatus strength, external-rotation strength, pain at rest, pain during activity, and symptom duration. For the ordinal model, postoperative satisfaction was analyzed on the full 0–4 scale.

**Supplementary Table S14.** Ordinal logistic regression sensitivity analysis for postoperative satisfaction using the full 0–4 scale, stratified by tear size

| **Factor** | **Small OR (95% CI)** | **p value** | **Medium OR (95% CI)** | **p value** | **Large OR (95% CI)** | **p value** |
| --- | --- | --- | --- | --- | --- | --- |
| Age | 1.03 (1.01–1.05) | 0.013 | 1.02 (1.00–1.03) | 0.032 | 1.00 (0.97–1.03) | 0.909 |
| Female sex | 0.94 (0.59–1.48) | 0.784 | 1.20 (0.87–1.66) | 0.274 | 0.88 (0.44–1.75) | 0.709 |
| Operative time (min) | 0.99 (0.96–1.01) | 0.297 | 1.00 (0.98–1.01) | 0.681 | 1.00 (0.98–1.03) | 0.791 |
| Number of anchors | 1.52 (1.04–2.23) | 0.031 | 1.03 (0.84–1.25) | 0.790 | 1.03 (0.72–1.47) | 0.880 |
| AP tear size (mm) | 1.00 (0.96–1.04) | 0.877 | 1.01 (0.98–1.03) | 0.625 | 1.02 (0.98–1.06) | 0.331 |
| Good tissue quality | 1.04 (0.34–3.14) | 0.949 | 1.04 (0.51–2.11) | 0.922 | 1.85 (0.68–5.03) | 0.231 |
| Preoperative stiffness | 0.89 (0.75–1.06) | 0.178 | 0.83 (0.71–0.97) | 0.021 | 1.17 (0.84–1.63) | 0.359 |
| Passive external rotation ROM (deg) | 0.99 (0.98–1.00) | 0.154 | 0.99 (0.99–1.00) | 0.231 | 1.00 (0.99–1.02) | 0.743 |
| Passive abduction ROM (deg) | 1.00 (1.00–1.01) | 0.095 | 1.01 (1.00–1.01) | 0.003 | 1.01 (1.00–1.02) | 0.165 |
| Preoperative supraspinatus strength | 0.99 (0.98–1.01) | 0.409 | 1.00 (0.99–1.01) | 0.503 | 0.98 (0.95–1.01) | 0.164 |
| Preoperative external rotation strength | 1.00 (0.99–1.02) | 0.599 | 1.01 (1.00–1.02) | 0.128 | 1.01 (0.99–1.04) | 0.375 |
| Pain at rest | 0.95 (0.75–1.19) | 0.638 | 0.88 (0.72–1.08) | 0.224 | 0.75 (0.50–1.13) | 0.171 |
| Pain during activity | 0.69 (0.46–1.04) | 0.076 | 0.85 (0.62–1.17) | 0.323 | 0.49 (0.21–1.17) | 0.109 |
| Symptom duration (days) | 1.00 (1.00–1.00) | 0.606 | 1.00 (1.00–1.00) | 0.888 | 1.00 (1.00–1.00) | 0.466 |

**Footnote.** Ordinal logistic models used the full 0–4 postoperative satisfaction scale and were adjusted for the same covariates as the main dichotomized satisfaction model.

**Supplementary Table S15.** Key coefficients from pooled ordinal logistic regression including tear size × number of anchors interaction

| **Term** | **OR (95% CI)** | **p value** |
| --- | --- | --- |
| Number of anchors (small tears reference) | 1.43 (1.03–1.99) | 0.032 |
| Interaction: medium × number of anchors | 0.72 (0.51–1.02) | 0.062 |
| Interaction: large × number of anchors | 0.74 (0.48–1.14) | 0.174 |

**Footnote.** Global likelihood-ratio test for the ordinal tear size × number of anchors interaction: χ² = 3.516, df = 2, p = 0.172.
